# The effects of short-term combined exercise training on telomere length in obese women: a prospective, interventional study

**DOI:** 10.1186/s40798-020-0235-7

**Published:** 2020-01-16

**Authors:** Camila Fernanda Cunha Brandao, Carla Barbosa Nonino, Flavia Giolo de Carvalho, Carolina Ferreira Nicoletti, Natalia Yumi Noronha, Rocio San Martin, Ellen Cristini de Freitas, Marcia Varella Morandi Junqueira-Franco, Julio Sergio Marchini

**Affiliations:** 1 Internal Medicine Department, Ribeirao Preto Medical School, University of Sao Paulo, Av. Bandeirantes 3900, Ribeirão Preto, 14049-900 Brazil; 2 Department of Health Sciences, Ribeirão Preto Medical School, University of São Paulo, São Paulo, Brazil; 3 School of Physical Education and Sport of Ribeirão Preto, University of São Paulo, São Paulo, Brazil

**Keywords:** Combined exercise training, Obesity, Physical Activity, Physical fitness, Telomere length, Waist circumference

## Abstract

**Background:**

Telomere length is inversely associated with the senescence and aging process. Parallelly, obesity can promote telomere shortening. Evidence suggests that physical activity may promote telomere elongation.

**Objective:**

This study’s objective is to evaluate the effects of combined exercise training on telomere length in obese women.

**Design and Methods:**

Twenty pre-menopausal women (BMI 30–40 kg/m^2^, 20–40 years) submitted to combined training (strength and aerobic exercises), but only 13 finished the protocol. Each exercise session lasted 55 min/day, three times a week, throughout 8 weeks. Anthropometric data, body composition, physical performance (Vo_2max_), and 8-h fasting blood samples were taken before and after 8 weeks of training. Leukocyte DNA was extracted for telomere length by RT-qPCR reaction, using the 2^−ΔΔCt^ methodology.

**Results:**

After the training intervention, significant differences (*p* < 0.05) were observed in telomere length (respectively before and after, 1.03 ± 0.04 to 1.07 ± 0.04 T/S ratio), fat-free mass (46 ± 7 to 48 ± 5 kg), Vo_2max_ (35 ± 3 to 38 ± 3 ml/kg/min), and waist circumference (96 ± 8 to 90 ± 6 cm). In addition, an inverse correlation between waist circumference and telomere length was found, before (*r* = − 0.536, *p* = 0.017) and after (*r* = − 0.655, *p* = 0.015) exercise training.

**Conclusion:**

Combined exercise promoted leukocyte telomere elongation in obese women. Besides, the data suggested that greater waist circumference may predict shorter telomere length.

**Clinical Trial Registration:**

ClinicalTrails.gov, NCT03119350. Retrospectively registered on 18 April 2017

## Key Points

- Combined physical training promotes telomere elongation in obese women.
- Waist circumference may influence telomere length in obese women after physical training.
- Short-term physical training increases physical performance in obese women independent of weight loss.

## Introduction

Telomeres are located at the end of linear eukaryotic chromosomes and had tandem sequences sheltered by a protein complex. These proteins promote telomere-ends protection, which leads to chromosomal stability and preserves genome information during replication processes [1]. Changes in telomere length (TL) are associated with cellular senescence [1], oxidative stress [2], increased inflammatory process [3], and DNA damage [4]. Moreover, telomere shortening is related to metabolic disorders and a sedentary lifestyle [2].

On the other hand, physical training is associated with improvements in many aspects of human health. These include better exercise capacity, endothelial function, and autonomic function and decreased high blood pressure, as well as a reduction of abdominal fat and decreased inflammatory parameters [5, 6]. Also, the beneficial effects of physical activity on cellular regeneration and senescence have been observed [3, 7, 8].

The aerobic exercise involves exercise performed for extended periods with extensive muscle activity involving consecutive repetitions that challenge the delivery of oxygen to the active muscles. Alternatively, strength or resistance exercise programs involve weight training or the use of high-resistance machines with exercise that is limited to a few repetitions before exhaustion [9]. In general, aerobic exercise induces more significant improvements in cardiorespiratory fitness and cardiometabolic variables, whereas resistance exercise mainly affects muscular strength and has beneficial effects on body composition [10]. A combination of endurance and resistance training could have an additive effect and is recommended for cardiovascular disease prevention in all subjects, including the obese subjects [6, 11].

Endurance training promotes longer telomere length in young athletes [7], while resistance training did not increase telomere length in young [12], and there are little data over the effects of combined exercise on TL. Thus, since subjects with obesity are more susceptible to telomere shortening, and previous studies [3, 6–8] suggest the benefits of endurance exercise on TL, the present study aimed to investigate the changes in TL of women with obesity before and after 8 weeks of a combined exercise training program and to identify possible correlations between body composition, anthropometric data, and TL.

## Methods and Procedures

### Participants and Study Design

Twenty volunteer females were selected by convenience sample, recruited among patients from Ribeirao Preto University Hospital, by disclosure in folders and social media. Inclusion criteria were as follows: age ranged from 20 to 40 years old, BMI between 30 to 40 kg/m^2^, sedentary lifestyle for at least 6 months prior to the study, and regular menses. Participants that reported a history of diabetes, hypertension, dyslipidemia, cancer, smoke, and any obesity-specific treatment (drugs or bariatric surgery) were excluded. Also, to avoid the possible biases due to hormonal influences, men were not included.

**Fig. 1:**
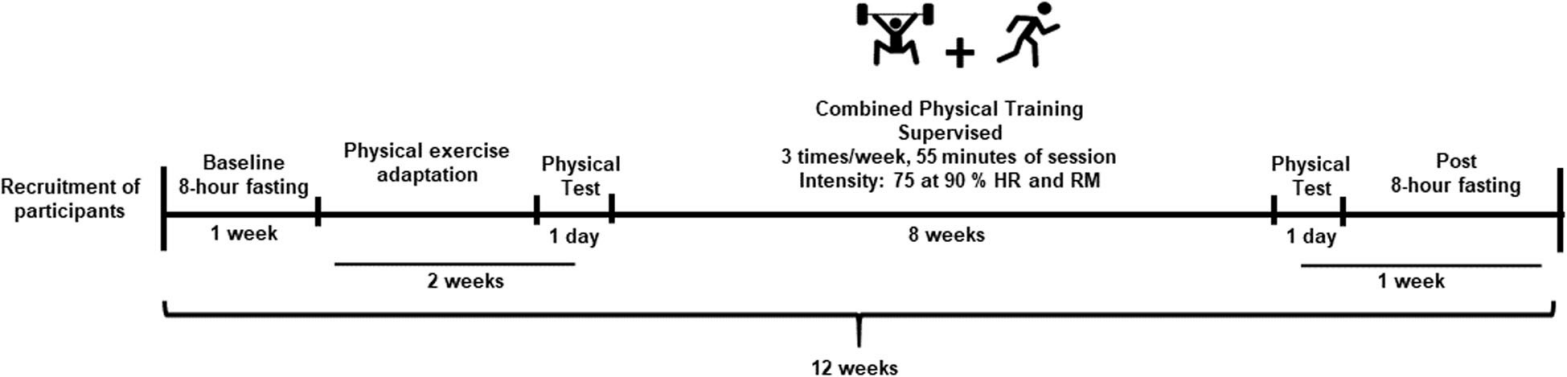
Experimental design. Evaluations and intervention of study for 12 weeks. HR, heart rate; RM, multiple repetitions. The intensity of exercise: 2 weeks of 75%, 4 weeks of 80%, 2 weeks of 90% from HRmax and RMmax

The prospective and controlled study lasted for 12 weeks, as shown in Fig. 1. Evaluations after 8-h fasting (anthropometric data, body composition, and blood collection) were done on the first week; adaptation to exercise and physical tests were done on the second and third weeks; intervention was done on the fourth to the 11th weeks; and in the 12th week, the first 2 days were for physical tests and after were for the blood collection (8-h fasting).

The intervention was at the University of São Paulo´s gym (Ribeirao Preto, SP, Brazil), 100% supervised, and with at least 50% attendance. The subjects, who completed the intervention, had an average 80% presence. The combined exercise training (alternating strength and aerobic exercise) consisted of 15 resistance exercises (for all the main muscle groups: chest, dorsum, biceps, triceps, deltoid, shoulders, quadriceps, gluteus, biceps femoral, rectus femoral, gastrocnemius, abdominal) for 30 s (it recommended that participants perform at least ten repetitions per exercise) alternated with 30 s of jogging [13]. The intervention had a duration of 8 weeks, with a frequency of three times/week with 55 min/day of duration and intensity of 75 to 90% of heart rate maximum (HR) and multiple repetitions maximum-RM (2 weeks of 75%, 4 weeks of 80%, and 2 weeks of 90% of HR and RM). The intensity of training was controlled by the heart rate monitor (Polar®) and the rating of perceived exertion (RPE) [14]. The same trained professional supervised all exercise sessions and the heart rate of participants. During the intervention, we emphasized to all participants to keep constant food intake. There were no diet intake restrictions.

The Research Ethics Committee of the Clinical Hospital from the Ribeirao Preto Medical School, University of São Paulo, approved the study (protocol # 1387.040/2016), and the study is in accordance with the standards of ethics outlined in the Declaration of Helsinki. All subjects gave free written consent.

### Phenotypic Measures

All subjects have fasted for 8 h for evaluation. Bodyweight was measured by an electronic platform Fiziola™ scale with a precision of 0.1 kg and a maximum capacity of 300 kg. A rigid vertical shaft with 0.5 cm graduation was used to measure body height. The waist circumference was measured with an inextensible tape at the largest circumference between the last rib and the iliac crest. The body composition was evaluated with the deuterium oxide dilution method [15], each volunteer having received a dose of 1 ml/kg, 7% deuterium oxide (Cambridge Isotope®, USA). Urine samples were collected before and 3 h after dose intake. Samples were stored at − 80 °C until analysis. Deuterium enrichment in urine samples was determined by mass spectrometry (Europa Scientific Hydra System™, Cheshire, United Kingdom).

Physical performance tests were performed before and after the intervention. The aerobic performance was evaluated by an adapted, incremental shuttle walking test [16]. It required the participants to walk/run up and down a 10-m course, which started at 4 km/h, increasing 0.28 m/s every 3 min by stage. The speed at which the participant walked/ran was dictated by an audio signal and was interrupted when subjects could not maintain the determined rhythm (Additional file 1: Table S1). The estimated maximum oxygen consumption (VO_2max_) was determined, according to Heyward [17].

The strength performance was evaluated by multiple repetitions (RM) in the bench press (upper limbs) and squat (lower limbs) exercise for determination of maximum force (kg). After warm-up, with determined load, each participant should perform the correct movement to exhaustion or to complete ten repetitions (if exhaustion was not achieved in ten repetitions, there was a rest of approximately 10 min for the next attempt). After the test, to calculate the maximum load or RM, we used the formula by Brzycki [18].

### Telomere Length Measures

After 8 h of fasting, a trained professional collected the patients’ peripheral blood in EDTA tubes. Genomic DNA was automatically extracted from the leukocyte using the Maxwell MDx (Promega Corporation®, Madison, WI) instrument and the Maxwell® Blood DNA Purification kit.

The relative quantification of TL was determined by telomere to single-copy gene ratio (T/S) from calculation: ΔC*t* (C*t*^(telomeres)^/C*t*^(single-gene)^). For the calculation of 2^−ΔΔCt^ in this assay, each sample was normalized to the average T/S ratio of a reference sample, using the standard curve and validation sample as the reference [19]. It measured by quantitative polymerase chain reaction (qPCR), according to [20]. The thermal cycler used was the 7500 Fast Real-Time PCR System (Applied Biosystems®).

For the reaction, the SYBR Green PCR Mastermix kit (Qiagen®) was used in a final volume of 20 μL. Concentrations of telomere repeat copy number (T) and 36B4 (Ribosomal Protein Large PO) (S) as reference for the single-copy gene were 700 nM of each primer. DNA concentrations were and 20 ng (these values were selected from the standard curve). Telomere primer sequences (5′ to > 3′) were tel 1, GGTTTTTGAGGGTGAGGGTGAGGGTGAGGGTGAGGGT and tel 2, TCCCGACTATCCCTATCCCTATCCCTATCCCTATCCCTA. Beta globin primer sequences were 36B4u, CCCATTCTATCATCAACGGGTACAA, and 36B4d, CAGCAAGTGGGAAGGTGTAATCC. For quality control, a minimum of three assays was performed, and the average was used for analysis [21, 22].

### Statistical Analysis

Descriptive statistics consisted of mean and standard deviation values. Data normality was verified by the Shapiro-Wilk test. Then, the appropriately paired *t* test or Wilcoxon test was used to compare data before and after interventions. To verify correlations, the Pearson or Spearman test was used. Associations were verified by linear regression. Statistical significance was considered at *p* < 0.05. All analysis was performed with SPSS™ version 20.0 software (SPSS Inc.).

## Results

Twenty women (34 ± 5 years and BMI of 34 ± 3 kg/m^2^) agreed voluntarily to be evaluated before starting the exercise training intervention. During the program, six subjects dropped out (two became ill, and four declined to finish the study without any specific reason). One subject was excluded because of technical issues related to leukocyte DNA extraction. Thus, 13 obese women were enrolled and finished the intervention.

After 8 weeks of combined exercise training, body weight and BMI did not change (*p* > 0.05). However, there is an increase of 6% in fat-free mass and a decrease of 2% in waist circumference (*p* < 0.05). Aerobic performance increased 8% of VO_2max_, 12% of the time, and 7% velocity of the physical test (*p* < 0.05). Strength performance (RM) by bench press was increased by 22% and squat by 138% (*p* < 0.05) (Table 1).

**Table 1:** Anthropometric and physical performance data of obese women before and after exercise training

Variables	Before (*n* = 20)	After (*n* = 13*)	*p*
Weight (kg)	89 ± 10	86 ± 9	0.254
BMI (kg/m^2^)	34 ± 3	33 ± 3	0.278
Waist circumference (cm)	96 ± 8	90 ± 6	*0.024*
Fat mass (kg)	43 ± 5	38 ± 7	0.080
Fat-free mass (kg)	46 ± 7	48 ± 5	*0.007*
V_O2_max (ml/kg/min)	35 ± 3	38 ± 3	*0.012*
Velocity (km/h)	7 ± 1	8 ± 1	*0.010*
Time (minutes)	13 ± 2	15 ± 2	*0.003*
RM bench press (kg)	29 ± 9	35 ± 10	*0.043*
RM squat (kg)	35 ± 19	68 ± 17	*0.001*

Data expressed as mean and standard deviation (M ± SD)

*BMI* body mass index, *V*_*O2*_*max* maximum oxygen consumption, *Velocity* velocity of last stage of shuttle walking test, *Time* total time of physical test, *RM Bench press* upper limb strength, *RM Squat* lower limb strength

*Six subjects from pre intervention gave up the study and one subject was excluded because of technical issues related to leukocyte DNA extraction

**Fig. 2:**
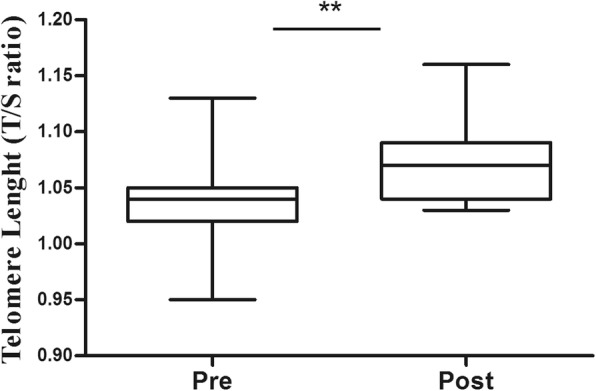
Telomere length, pre- and post-8 weeks exercise training, of women with obesity. *p < 0.05

**Fig. 3:**
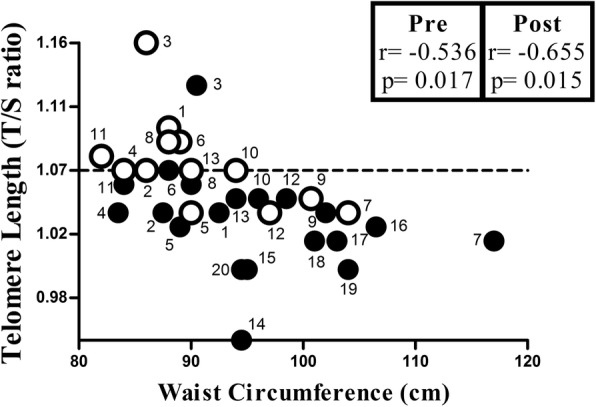
Correlation between waist circumference and telomere length. Black symbols: pre-intervention (*n* = 20 subjects). White symbols: post-intervention (*n* = 13 subjects). Numbers of symbols represent each subject

TL increased by 2% after exercise training (from 1.03 ± 0.04 to 1.07 ± 0.04, *p* = 0.001) (Fig. 2). Moreover, we found an inverse correlation between waist circumference and TL, before (*r* = − 0.536, *p* = 0.017, *r*^2^ = 0.117) and after (*r* = − 0.655, *p* = 0.015, *r*^2^ = 0.321) exercise training (Fig. 3).

## Discussion

As the main results, we found that the 8-week combined exercise training program increased telomere length, physical performance, and fat-free mass, while it decreased waist circumference. Our results showed that subjects with bigger waist circumference have lower telomere length.

Previous studies have shown the potential benefits of exercise for obesity treatment due to its effects on energy expenditure, modulation of body composition, and enhancement of physical performance [23–25]. Additionally, previous studies have shown the potential relationship between higher physical activity levels and longer TL in leukocytes [25–27] and skeletal muscle cells [28, 29]. Besides that, it has shown that exercise leads to a regulation of telomerase expression [30]. However, different training modalities exert differential cellular effects. A study showed that endurance and interval training increased TL, while resistance training did not change TL [12].

Available data on the relationship between obesity and TL in adults have shown controversial results. However, some studies reported an inverse association of leukocytes TL with obesity [31–33]. Higher total and abdominal adiposity have lower TL in 309 subjects [31]. In elderly subjects, it was found that shorter TL may be a risk factor for increased adiposity [32], even it is found in 521 elderly subjects that TL is inversely associated with changes in obesity parameters [33]. However, other studies did not find it in 2509 subjects [34] and others in 317 subjects [35], and there are no linear associations with cardiovascular diseases. It is worthy to note that all these studies were from the community without any specific intervention. Likewise, a study with post-menopausal women did not change TL after exercise and diet intervention [36].

Some studies found longer telomere and weight loss in subjects with obesity [37–39]. Moreover, the authors did not find an association between the magnitude of weight loss after diet plus exercise intervention and TL [36]. In the present study, although we did not observe significant weight loss after the intervention, we detected a TL increase. Thus, total body weight loss may not be a significant predictive effect regarding TL elongation. In line with this, a study with green tea supplementation for 8 weeks for women with obesity also did not find changes in body weight. However, it observed that supplementation promotes TL increase and a positive association between BMI and shorter TL [22].

Inflammation and oxidative stress related to the obesity condition are factors that accelerate TL shortening [40]. The fact is that physical exercises can regulate antioxidant defense systems reducing DNA damage and chronic systemic inflammation per se. Independent of weight loss, it may explain our major results that physical training intervention protects against telomere attrition.

Higher waist-to-hip ratio is considered an independent predictor of TL shortening, and abdominal obesity appeared to have a stronger effect on telomere reduction [41]. Indeed, body fat mass percentages correlate with telomere shortening [42]. In our study, we found that waist circumference can influence telomere shortening, influenced by physical training.

The shortening of the DNA component of TL may induce genomic instability, cellular senescence, and apoptosis and, as a result, may facilitate cardiovascular disease risk [1]. Subjects who engaged in more physical activity sat less and had higher cardiorespiratory fitness and longer TL [43]. A study that compared the differential effects of endurance, interval, and resistance training found that resistance training did not increase telomerase activity and TL [7]. Although fewer studies have shown the effects of resistance training on TL, our data showed some effects. The resistance and endurance exercises could be a strategy to promote TL elongation and increase physical performance capacity. Therefore, our results also demonstrated that the benefits of the association of aerobic and strength exercise go further than weight and body composition management.

Shorting telomere length is associated with early elderly processing. Besides, telomere length is lower in people with obesity [2]. Parallel, telomere length is elongated in physically active people, and exercise training changes body composition [3].

The relatively limited sample size due to the peculiar intervention design of the study is a limitation of the present study. However, this trial is a prospective, controlled, and supervised training study. To keep subjects on the intervention and control heart rate and ambient (temperature and humidity) is expensive and complicated.

## Conclusion

The short-term combined exercise training (8-weeks) promoted TL elongation, an increase of free-fat mass, an increase of physical performance, and waist circumference reduction. There is a negative correlation between TL and waist circumference.

Although this time of training was unable to promote a significant weight loss, this manuscript has shown metabolic benefits from physical activity to obesity, for example, enhanced physical fitness and fat-free mass. It is crucial to the point that exercise training may prevent comorbidities associated with obesity due enhance physical fitness. Also, it may prevent the shortening of telomere promoted by obesity. Furthermore, the abdominal adiposity evaluated by waist circumference is negatively related to TL.

## Supplementary information

**Additional file 1: Table S1.** Shuttle walking test – Increments of velocity, time and numbers of courses from test

## Data Availability

All data may be made available from the corresponding author upon reasonable request.

## Abbreviations

BMI: Body mass index
qPCR: Quantitative polymerase chain reaction
RM: Multiple repetitions
RPE: Rating of perceived exertion
TL: Telomere length
VO_2max_: Maximum oxygen consumption
