# Supplementary material for: The effects of short-term combined exercise training on telomere length in obese women: a prospective, interventional study
Source: Sports Med Open. 2020 Jan 16;6:5. doi: 10.1186/s40798-020-0235-7 (PMC6965549; doi:10.1186/s40798-020-0235-7)
Supplement: Supplementary file 1 — Additional file 1: Table S1. Shuttle walking test – Increments of velocity, time and numbers of courses from test [file 40798_2020_235_MOESM1_ESM.docx]

**Electronic Supplementary Material (ESM) file:** The effects of short-term combined exercise training on telomere length in obese women: a prospective, interventional study; Sports Medicine - Open; Camila Fernanda Cunha Brandao, Carla Barbosa Nonino, Flavia Giolo de Carvalho, Carolina Ferreira Nicoletti, Natalia Yumi Noronha, Rocio San Martin, Ellen Cristini de Freitas, Marcia Varella Morandi Junqueira-Franco, Julio Sergio Marchini; *Division of Nutrology, Ribeirão Preto Medical School, University of São Paulo, Brazil;* camilafbrandao@hotmail.com

| **Supplementary Table 1.** Shuttle walking test – Increments of velocity, time and numbers of courses from test | | | |
| --- | --- | --- | --- |
| **Km/h** | **Time (seconds) / course** | **Courses 10 meters numbers** | **Total time** |
| 4 | 9.0 | 20 | 3 minutes |
| 5 | 7.2 | 25 | 3 minutes |
| 6 | 6.0 | 30 | 3 minutes |
| 7 | 5.1 | 35 | 3 minutes |
| 8 | 4.5 | 40 | 3 minutes |
| 9 | 4.0 | 45 | 3 minutes |
| 10 | 3.6 | 50 | 3 minutes |
| 11 | 3.3 | 55 | 3 minutes |
| 12 | 3.0 | 60 | 3 minutes |
| 13 | 2.8 | 65 | 3 minutes |
| 14 | 2.6 | 70 | 3 minutes |
| 15 | 2.4 | 75 | 3 minutes |
| Test adapted by Singh [13] | | | |
